# Low transplantation rate in the contemporary Fontan cohort: A marker of success or a reflection of unmet need?

**DOI:** 10.1016/j.xjon.2026.102049

**Published:** 2026-08-06

**Authors:** Katherine Kearney, S. Lucy Roche, Peter Macdonald

**Affiliations:** a Department of Cardiology, UT Southwestern Medical Center, Dallas, Tex; b Peter Munk Cardiac Centre, University Health Network, Toronto, Ontario, Canada; c Department of Medicine, University of Toronto, Toronto, Ontario, Canada; d The Labatt Family Heart Centre, The Hospital for Sick Children, Toronto, Ontario, Canada; e Heart Transplant Unit, St Vincent's Hospital Sydney, Darlinghurst, New South Wales, Australia; f Victor Chang Cardiac Research Institute, Sydney, New South Wales, Australia; g School of Medicine, University of New South Wales, Sydney, New South Wales, Australia

To the Editor:

**Figure:**
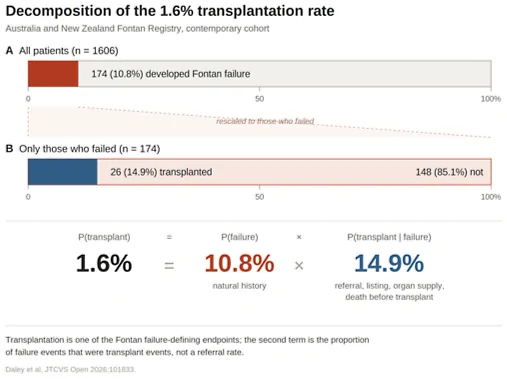

We read with interest the contemporary outcomes of Daley and colleagues^1^ from the Australian and New Zealand Fontan Registry. The survival data reflect genuine advances in surgical care. However, the reported 1.6% transplantation rate, framed in public commentary as a marker of success, merits scrutiny, not celebration.

The core paradox is arithmetical: Fontan failure occurs in 26% to 30% of patients by 20 years,^2^ yet transplantation occurred in only 1.6% of this cohort. The gap will widen: hypoplastic left heart syndrome, the Fontan subgroup with the greatest complexity, increased from 3% of the 1988-1999 era cohort to 32% of those operated between 2020 and 2024.^2^ This increasing burden makes a low transplantation rate harder, not easier, to read as reassuring.

The strongest evidence for access failure is within this registry itself. Shi and colleagues^3^ found patients followed outside the national referral center were nearly 5 times less likely to undergo transplantation (hazard ratio, 4.7; 95% CI, 1.7-13.5), with cumulative rates of 6.8% versus 1.2% at 20 years. Same disease, different geography; a 4-fold gap reflecting referral, not biology.

This pattern is not unique to Australian and New Zealand. The authors of a Fontan cohort from the United Kingdom reported 46 adverse events: 44 deaths and 2 transplantations.^4^ Yet transplantation for the failing Fontan is achievable: another group from the United Kingdom reported good posttransplant survival, with referral timing, not feasibility, as the barrier.^5^ This recurring pattern points to a structural, not national, failure to refer.

The present paper itself quantifies this cost. Of 99 patients who developed Fontan failure without death or transplant as the first event, survival was only 75% at 10 years and 60% at 15 years.^2^ More than one half of these patients experienced New York Heart Association class III-IV heart failure, generally considered a Class 1a indication for transplant referral. These are not outcomes consistent with a system that is managing Fontan failure adequately. The bottleneck would appear to be referral, not surgery—but only patients who are referred before end-organ injury becomes irreversible will benefit.

We urge future registry analyses to address 3 gaps in our knowledge: transplant referral and waitlist rates, not solely completion rates; cause-specific mortality disaggregated by transplant access; and functional trajectory in patients who die without specialist evaluation. A 1.6% transplantation rate cannot be read as success without knowing how many of the remainder died of Fontan failure that was never referred.

## Conflict of Interest Statement

The authors reported no conflicts of interest.

The *Journal* policy requires editors and reviewers to disclose conflicts of interest and to decline handling or reviewing manuscripts for which they may have a conflict of interest. The editors and reviewers of this article have no conflicts of interest.
